# A comparable method to Gd-contrast enhancement in the preoperative evaluation of anal fistula

**DOI:** 10.1097/MD.0000000000017807

**Published:** 2019-11-01

**Authors:** Chao Gu, Yu Wang, Lixia Lai, Weiwei Han, Jiansheng Li, Haichang Xing, Yongjun Huo, Chuanting Li, Keyun Bai

**Affiliations:** a Affiliated Hospital of Shandong University of Traditional Chinese Medicine, Jinan, Shandong; b China-Japan Friendship Hospital, Beijng; c Shandong Medical Imaging Research Institute, Jinan, Shandong, China.

**Keywords:** anal fistula, diffusion, magnetic resonance imaging, morphology

## Abstract

To explore a comparable method to Gd-contrast enhancement in the preoperative evaluation of anal fistula to evaluate its morphology changes.

Forty-six patients with anal fistula were enrolled. Each patient acquired a 3.0T magnetic resonance imaging (MRI) routine sequence, diffusion-weighted imaging (DWI) sequence and fat suppression T1 weighted imaging (FS T1WI) contrast enhancement (CE) scanning. To record the morphology performances of the internal orifice and the fistulas on the transverse images of fat suppression T2 weighted imaging (FS T2WI), DWI, FS T2WI combined with DWI, FS T1WI Gd-CE, with the standard of the surgical pathology results. Two observers evaluated images in consensus. The conspicuity and the diagnostic performance rate were compared between the 4 imaging data sets.

The consistencies of interobservers about the conspicuity scores and the diagnostic performance rates of the internal orifice and the fistula were good. The conspicuity of the internal orifice was higher for the set of FS T2WI, FS T2WI+DWI, and FS T1WI+CE than DWI. The diagnostic performance rate of the internal orifice was higher for the set of FS T2WI, FS T2WI+DWI, and FS T1WI+CE than DWI. The conspicuity of the fistula was higher for the set of FS T2WI+DWI and FS T1WI+CE than FS T2WI or DWI. There were no significantly differences between the 4 sets of FS T2WI, DWI, FS T2WI+DWI, and FS T1WI+CE in the diagnostic performance rate of the fistula.

The set of FS T2WI combined with DWI was comparable to FS T1WI CE in evaluation of anal fistula morphology changes.

## 1 Introduction

Anal fistula refers to the anomalous connection between the anus or rectum and the skin around the anus with the granulation tissue lining the anal fistula,^[1]^ which is the typical result of the healing of perianal abscess. It occurs to patients of any age and the average age is between 20 and 40. The incidence of anal fistula is high^[2,3]^ while the clinical cure rate is relatively low, which is attributed to the hidden secondary fistula or small abscess being ignored.

Magnetic resonance imaging (MRI) is currently recognized as the best method for detecting and evaluating the details of the anal fistula.^[4]^ It is demonstrated that the postoperative complications of anorectal fistula could be decreased by about 75% thanks to MRI examination.^[5]^

The great significance of the 3 imaging data sets has been emphasized in the literature including the T2 weighted imaging (T2WI),^[6]^ fat suppression T2 weighted imaging (FS T2WI)^[7,8]^ and fat suppression T1 weighted imaging contrast-enhanced (FS T1WI+CE) sequences.^[9]^ However, the use of contrast agent is relatively contraindicated in patients with renal insufficiency result from the increased risk of renal systemic fibrosis.^[10]^ And multiple studies have shown that repeat administration of the gadolinium may result in the deposition in the deep nuclei of the brain.^[11,12]^ It is critical for looking for a noninvasive method to assess anal fistula. In another hand, there was no report about the comparison between FS T2WI+DWI and either FS T2WI or DWI alone and the comparison between FS T2WI+DWI and FS T1WI+CE in the literature. Therefore, the purpose of this study is to compare the lesion conspicuity and diagnostic performance rate of four imaging data sets (FS T2WI, diffusion-weighted imaging (DWI), FS T2WI combined with DWI[FS T2WI+DWI], FS T1WI+CE) in assessing perianal fistulas, so as to explore a comparable method to Gd-contrast enhancement in the preoperative evaluation of anal fistula.

## 2 Materials and methods

### 2.1 Study population

From January 2017 to December 2018, 323 patients were enrolled in the study who had been clinically diagnosed perianal fistulas with detailed surgical and pathology reports. The inclusion criteria were as follows:

(1) Patient who underwent magnetic resonance (MR) imaging before surgery and
(2) Patient without operation history because of perianal fistula.

A total of 52 patients were selected. Six patients were excluded for unavailable DWI (n = 3), FS-T2WI (n = 2) or FS T1WI+CE (n = 1). Thus, 46 patients (39 males and 7 females) were confirmed in the final study, aged 18 to 80 years (mean 41.67 ± 2.18, with a median of 39.5 years), complaining of the perianal swelling, fever or pain. There was a fistula or a hard knot around the anus. And sometimes purulent or bloody discharge could be seen. With the consent of the hospital ethics committee, all patients were informed of the condition before examination, and the informed consent was signed by the patient or legal guardian.

### 2.2 Techniques and methods

All the patients underwent MRI scanning within 1 week before surgical treatment, and were confirmed as anal fistula by the surgery and pathology. All patients were scanned with 3.0T Siemens MR scanner (Siemens, Skyra 3.0T), covered with 18-channel body coil, in supine position with head first, with the symphysis pubis as the center. No intestinal preparation was required.

The MRI protocols contained axial T1-weighted imaging turbo spin echo (TSE), coronal T2-weighted imaging, axial and sagittal T2-weighted imaging with fat suppression, followed by DWI. Finally, the volumetric interpolated breath-hold examination (VIBE) acquisition technology was used to conduct T1-weighted with fat suppression sequence before and after intravenous gadolinium administration (gadodiamide, 0.1 mmol/kg, with the injection rate of 2.5 ml/second, and 20 ml saline injected at last). The main parameters were as follows: turbo spin echo (TSE) T1-weighted imaging (T1WI): axial; repetition time (TR)/echo time (TE), 600/18 ms; field of view (FOV), 280 mm × 280 mm; matrix, 314 × 448, slice thickness, 4 mm; and number of slices, 24. TSE T2-weighted imaging (T2WI) with spectral adiabatic inversion recovery (SPAIR) for fat suppression: axial; TR/TE, 3320/85 ms; FOV, 280 mm × 280 mm; matrix, 307 × 384; slice thickness, 4 mm; and number of slices, 24. T2-weighted imaging (FS-T2WI): sagittal; TR/TE, 5450/86 ms; FOV, 260mm × 260 mm; matrix, 320 × 240; slice thickness, 4 mm; and number of slices, 25. DWI (spin echo-echo-planar imaging, SE-EPI): axial; TR/TE, 5800/76 ms; FOV, 320 mm × 320 mm; matrix, 118 × 168; slice thickness, 5 mm; number of slices, 25; NSA, 1; b = 0.800 s/mm^2^.

### 2.3 Imaging analysis and interpretation

After the scanning, the images were transmitted to the picture archiving and communication system (PACS). The surgical pathology results were taken as the standard. Two physicians with more than 8 years’ experience in MR diagnosis analyzed and evaluated the images independently. After the initial independent analyses, the results were discussed by the 2 observers and modified if necessary, applying a consensus reading method. Recording the detection of internal orifice, main fistula, secondary or branch fistula or abscess of anal fistula on the transverse images of FS T2WI, DWI, FS T2WI+DWI, FS T1WI+CE. According to the Parks classification,^[13]^ the anal fistula could be divided into 4 types. According to 4-point scale in the references by Hori,^[14]^ and the definition, contour and margin of the internal orifice and fistula, and the relationship between them with the sphincters, scored each sequence on the conspicuity and diagnostic performance rate of the internal orifices and fistulas on a 4-point scale: 1, probably not a fistula or an internal orifice; 2, uncertain or indistinct; 3, possible a fistula or an internal orifice but obscure; and 4, definite and legible fistula or an internal orifice.

### 2.4 Statistical analysis

Statistical analysis was done with the software package SPSS 25.0. Kappa conformance test was performed on the 2 observers: k ≤ 0.4, poor consistency; 0.4 < k ≤ 0.6, medium consistency; 0.6 < k ≤ 0.8, good consistency; k > 0.8, very good consistency. The diagnostic performance rate of the anal fistula was compared with chi-square test; the conspicuity scores of the internal orifices and fistulas were compared with rank test. A *P* value of less than .05 was considered to indicate a statistically significant difference. Bonferroni correction (n = 4) was used for multiple comparisons, a *P* value of less than .008 ((2 × 0.05)/4 (4–1)) was considered to indicate a statistically significant difference.

## 3 Results

### 3.1 Classification of fistulas and the MR signal

In 46 cases of anal fistula, a total of 51 internal orifices, 62 primary fistulas and 34 abscesses were found in surgery, with 2 internal orifices found in 5 patients, and 3 horseshoe fistulas. According to the classification system of Parks, the perianal fistulas consisted of 22 intersphincteric fistulas (47.8%), 20 transsphincteric fistulas (43.5%), 3 suprasphincteric fistulas (6.5%) and 1 extrasphincteric fistula (2.2%).

### 3.2 The consistency of the 2 observers of the internal and fistulas in each set

The consistencies of interobservers about the conspicuity score of the internal orifice of anal fistula (FS T2WI, k = 0.816; DWI, k = 0.799; FS T1WI+CE, k = 0.828; FS T2WI+DWI, k = 0.821), the diagnostic performance rate of the internal orifice (FS T2WI, k = 0.940; DWI, k = 0.958; FS T1WI+CE, k = 0.852; FS T2WI+DWI, k = 0.812), and the conspicuity score of the fistula (FS T2WI, k = 0.812). T2WI, k = 0.824; DWI, k = 0.882; FS T1WI+CE, k = 0.718; FS T2WI+DWI, k = 0.851), the diagnostic performance rate of the fistula (FS T2WI, k = 0.783; DWI, k = 0.816; FS T1WI+CE, k = 0.641; FS T2WI+DWI, k = 0.792) were normal.

### 3.3 The comparison of the conspicuity score and diagnostic performance rate of the internal orifice in each set

The conspicuity of the internal orifice was higher for the set of FS T2WI, FS T2WI+DWI and FS T1WI+CE than that of DWI (*P* = .000263, .000002, .000155, respectively). However, there was no significant differences between the sets of FS T2WI, FS T2WI+DWI, and FS T1WI+CE (Table 1).

**Table 1:**
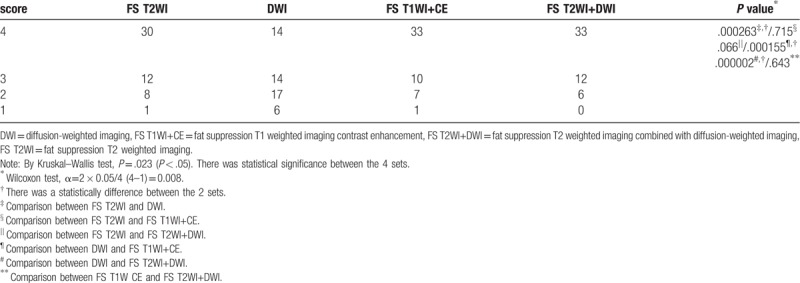
Comparison of the conspicuity score of the internal orifice in each set.

The diagnostic performance rate of the internal orifice was higher for the set of FS T2WI, FS T2WI+DWI, and FS T1WI+CE than DWI (*P* = .003, .001, .000190, respectively). However, there was no significant between the sets of FS T2WI, FS T2WI+DWI, and FS T1WI+CE (Table 2).

**Table 2:**
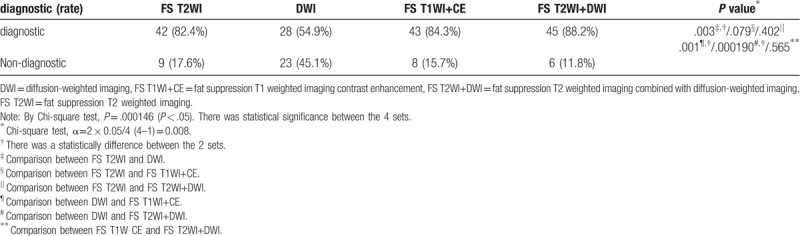
Comparison of the diagnostic performance rate of the internal orifice in each set.

### 3.4 The comparison of the conspicuity score and diagnostic performance rate of the fistula in each set

**Figure 1:**
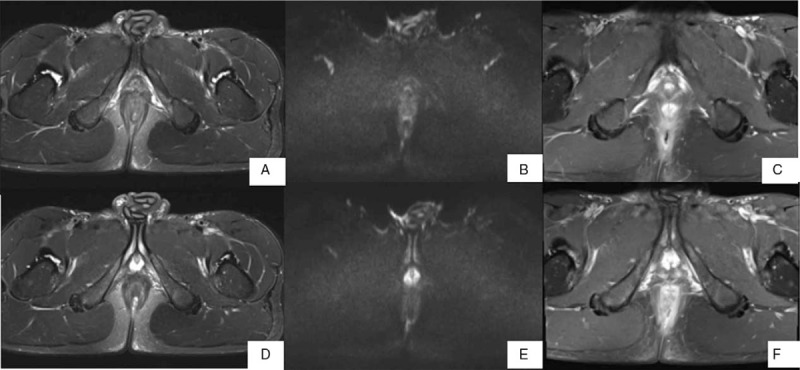
MR images of a 27-year-old man with an intersphincteric perianal fistula. (A, D) Axial fat suppressed T2-weighted imaging (FS T2WI), and (B, E) axial diffusion-weighted imaging (DWI) show the internal orifice (thin arrow) and the fistula tract (thick arrow) respectively. (C, F) The internal orifice is invisible on axial fat suppressed T1-weighted imaging with contrast enhanced (FS T1WI+CE), and the fistula tract (thick arrow) is nebulous because the comparative enhancement of the fistula tract and the inflammatory structure around the fiatula.

**Figure 2:**
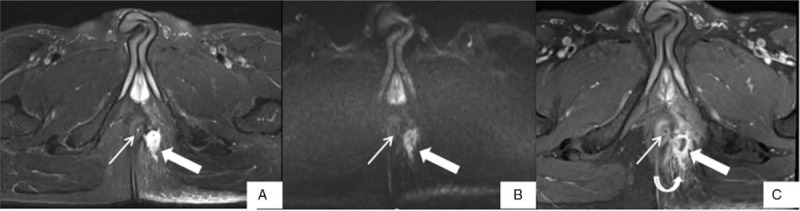
MR images of a 49-year-old man with a transsphincteric perianal fistula. (A) The internal orifice (thin arrow) is identified in the 3 o’clock direction, and the fistula tract (thick arrow) is noted in the left ischioanal fossa on axial fat suppressed T2-weighted imaging (FS T2WI). (B) The internal orifice (thin arrow) is almost invisible on axial diffusion-weighted imaging (DWI), and the fistula tract (thick arrow) is noted in the left ischioanal fossa. (C) The internal orifice (thin arrow) is identified in the 3 o’clock direction, and the fistula tract (thick arrow) is noted in the left ischioanal fossa on axial fat suppressed T1-weighted imaging with contrast enhanced (FS T1WI+CE). It shows not only the enhancement of the fistula tract, but also the enhancement of the inflammatory infection tissue (curved arrow) around the fistula. It is recorded a slightly higher confidence score for lesion conspicuity on FS T2WI than on FS T1WI+CE.

The conspicuity of the fistula was higher for the set of FS T2WI+DWI and FS T1WI+CE than that of FS T2WI or DWI. However, there was no significant differences between the sets of FS T2WI+DWI and FS T1WI+CE (Table 3, Figs. 1 and 2).

**Table 3:**
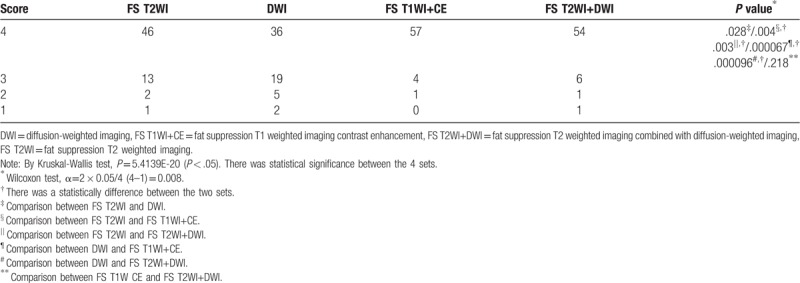
Comparison of the conspicuity score of the fistula in each set.

There were no significantly differences between the four sets of FS T2WI, DWI, FS T2WI+DWI, and FS T1WI+CE in the diagnostic performance rate of the fistula (Table 4).

**Table 4:**
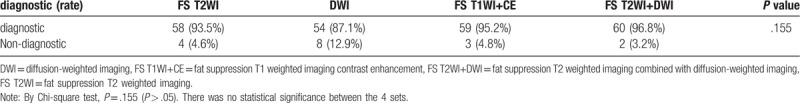
Comparison of the diagnostic performance rate of the fistula in each set.

## 4 Discussion

Anal fistula is an abnormal connection between the perianal skin and the anal or rectal canal. It is mainly composed of the internal orifice, fistula and external orifice. The diagnosis and treatment of internal orifice and fistula is critical to decide the success of operation. However, there was few literatures about the clarity of the internal orifice. Most of the internal orifices are found in the level of the dentate line of the anas, and are commonly seen in the back of the anal canal, at 6 o’clock in the bladder lithotomy position.^[15]^ Although it is difficult to visualize the dentate line as an integral anatomical structure, we can still determine its general position by MRI. The dentate line is located approximately in the middle of the anal canal between the upper edge of the puborectal muscle and the lower skin of the sphincter, which can be positioned accurately at the axis when combined with coronal images.

The relationship between the morphology of the fistula and the sphincter is another factor closely related to the surgery. The reason of fistulas recurrence is usually because of the neglecting of small hidden fistula during the operation. Therefore, it is necessary to obtain accurate information about the fistula before operation.

**Figure 3:**
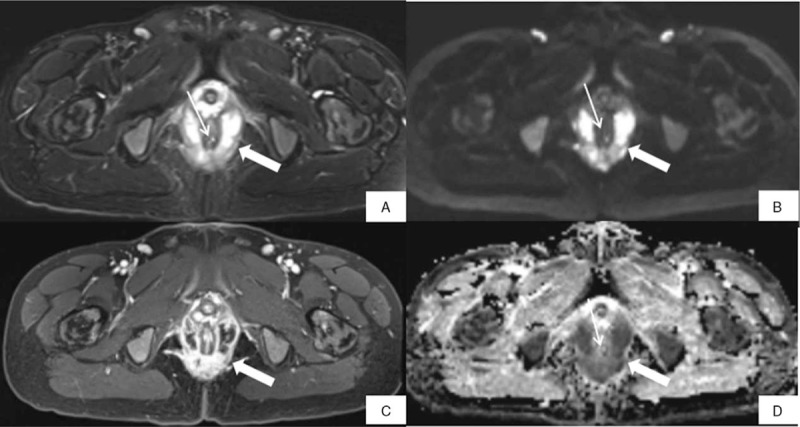
MR images of a 23-year-old man with a horseshoe fistula. (A) The internal orifice (thin arrow) is identified in the 5 o’clock direction, and the fistula tract (thick arrow) is noted around the anal canal on axial fat suppressed T2-weighted imaging (FS T2WI). (B, D) The internal orifice (thin arrow) and fistula tract (thick arrow) are both obviously showed on axial diffusion-weighted imaging (DWI) and the corresponding map of apparent diffusion coefficient (ADC). (C) The internal orifice is not identified on axial fat suppressed T1-weighted imaging with contrast enhanced (FS T1WI+CE), which maybe the result of the enhancement of the internal orifice and the mocosa at the same time. The fistula tract is visible (thick arrow).

It have been shown in the study that the detection rate of anal fistula by MRI is about 82.7% to 97%,^[15,16]^ and the highest rate of 97%^[15]^ was the result from the application of the endorectal coil. Kulvinder Singh et al^[17]^ showed that the accuracy of FS T2WI was 91%, FS T1WI + CE was 85% in 45 patients with anal fistula preoperative MRI. Jiyeon Baik et al^[6]^ compared the diagnosis rate of internal orifice of 24 cases of anal fistula by 2 observers. For observer 1, the display rate of internal orifice by T2WI combined with DWI (96%) was slightly higher than that by FS T1WI CE (92%), while for observer 2, FS T2WI+DWI (88.2%) are slightly higher than FS T1WI+CE (84.3%). In general, there was no difference between T2WI and DWI (96%) and FS T1WI+CE (96%). Our results showed that FS T2WI+DWI (88.2%), FS T1WI+CE (84.3%), FS T2WI (82.4%), and DWI (54.9%). FS T2WI+DWI, FS T1WI +CE, and FS T2WI were superior to DWI, respectively, but there was no significant difference between the first three sets. Hori et al evaluated 20 fistulas in 13 patients by FS T2WI, DWI, and FS T1WI+CE,^[14]^ which demonstrated that there were no differences between DWI combined with FS T2WI (95%) and FS T1WI+CE combined with FS T2WI (95%) in the fistula display rate and both were superior to FS T2WI (90%). In theory, when the fistula directly extends to the mucous membrane of the anal canal, it is easy to confirm the location of internal orifice. However, the circumferential thickness of the anas and the mucosa of the internal sphincter are not uniform in normal people actually. Sometimes local thickening of the mucosa shows high signal on FS T2WI sequence. Small vessels around the anas can also show high signal like dot or strip, which are similar to the signal of internal orifice. It is often difficult to distinguish. Although contrast enhancement (CE) can improve the detection rate of the internal orifice, it is sometimes difficult to distinguish from the perianal blood vessels or mucosa. However, the combination of DWI sequence becomes essential to increase the diagnostic accuracy (Fig. 3).

Our study may have some limitations. First, our retrospective study had a relatively low number of samples. Therefore, we need to expand the samples for further prospective investigation, while the imaging findings and conclusions should be interpreted as preliminary. Second, in order to improve the reliability of scoring and enhance the accuracy of diagnosis, we performed the scanning by multiple sequences and multiple directions in the examination of anal fistula, so it is unavoidable to combine other sequences, and the double-blind method was not used strictly to evaluate the single sequence. Future studies should take care to this point.

## 5 Conclusion

The conspicuity and diagnostic performance rate of FS T2WI combined with DWI were comparable to that of FS T1WI CE. The combination of FS T2WI and DWI could be the best sequence for noninvasive evaluation of anal fistula morphology changes. MRI could assess the morphology changes of anal fistula accurately, which is of great guiding significance for the treatment of anal fistula.

## Author contributions

**Conceptualization:** Chao Gu, Yu Wang, Weiwei Han, Jiansheng Li, Haichang Xing, Chuanting Li, Keyun Bai.

**Data curation:** Chao Gu, Yu Wang, Lixia Lai, Weiwei Han, Jiansheng Li, Haichang Xing.

**Formal analysis:** Yu Wang, Keyun Bai.

**Investigation:** Chao Gu, Yu Wang, Lixia Lai, Jiansheng Li, Keyun Bai.

**Methodology:** Chao Gu, Yu Wang, Lixia Lai, Weiwei Han, Jiansheng Li, Haichang Xing, Yongjun Huo, Chuanting Li, Keyun Bai.

**Project administration:** Chao Gu, Yu Wang, Haichang Xing, Yongjun Huo, Chuanting Li, Keyun Bai.

**Resources:** Weiwei Han, Jiansheng Li, Haichang Xing, Yongjun Huo, Keyun Bai.

**Supervision:** Chao Gu, Yu Wang, Lixia Lai, Weiwei Han, Haichang Xing, Yongjun Huo, Keyun Bai.

**Writing – original draft:** Yu Wang, Lixia Lai, Weiwei Han, Haichang Xing, Keyun Bai.

**Writing – review & editing:** Chao Gu, Chuanting Li, Keyun Bai.
